# The Correlation of CD206, CD209, and Disease Severity in Behçet's Disease with Arthritis

**DOI:** 10.1155/2017/7539529

**Published:** 2017-03-09

**Authors:** Bunsoon Choi, Chang-Hee Suh, Hyoun-Ah Kim, Hasan M. Sayeed, Seonghyang Sohn

**Affiliations:** ^1^Department of Microbiology, Ajou University School of Medicine, Suwon, Republic of Korea; ^2^Department of Rheumatology, Ajou University School of Medicine, Suwon, Republic of Korea; ^3^Department of Biomedical Sciences, Ajou University School of Medicine, Suwon, Republic of Korea

## Abstract

The purpose of this study was to clarify the role of pattern recognition receptors in Behçet's disease (BD). The frequencies of several pattern recognition receptors (CD11b, CD11c, CD32, CD206, CD209, and dectin-1) were analyzed in patients with BD by flow cytometry, and cytokine levels, interleukin- (IL-) 18, IL-23, and IL-17A, were compared in plasma. The analysis was performed in active (*n* = 13) and inactive (*n* = 13) stages of BD patients. Rheumatoid arthritis patients (*n* = 19), as a disease control, and healthy control (HC) (*n* = 19) were enrolled. The frequencies of CD11b+ and CD32+ cells were significantly increased in active BD patients compared to HC. Disease severity score was correlated to CD11c+, CD206+, and CD209+ in whole leukocytes and CD11b+, CD11c+, CD206+, CD209+, and Dectin-1+ in granulocytes. The plasma levels of IL-17A were significantly different between HC and active BD. IL-18 showed significant difference between active and inactive BD patients. From this study, we concluded the expressions of several pattern recognition receptors were correlated to the joint symptoms of BD.

## 1. Introduction

In immune dysfunction of Behçet's disease (BD), innate immunity is regarded to become more significantly involved in the pathogenesis. The important function of innate immunity is the initiation of defense against infection, such as virus, bacteria, and fungus, and linking to the adaptive immune responses [1]. Pattern recognition receptors (PRR) are proteins expressed on the cells of the innate immune system [2]. PRR can recognize pathogen-associated molecular patterns [3]. Most classes of the human pathogens are recognized by c-type lectin receptors (CLR), which is one kind of PRR [4]. CLR includes the mannose receptor (CD206), primarily present on the surface of macrophages and dendritic cells (DC), and asialoglycoprotein receptor family which includes DC-specific intercellular adhesion molecule-3-grabbing nonintegrin (CD209) and DC-associated C-type lectin-1 (Dectin-1) [5].

Several chronic inflammatory diseases, such as colitis, Crohn's disease, Kawasaki disease, and rheumatoid arthritis (RA), have been reported to be significantly associated with CD206, CD209, and Dectin-1 [6–8]. However, in BD, the correlation of CLR has not been published at all except mannose-binding lectin, one kind of soluble protein of CLR [9]. Therefore, in this study, the expression of CD206, CD209, and Dectin-1 was analyzed and compared between active and inactive BD patients with arthritis. The frequencies of CD11b, CD11c, and CD32 were also analyzed by combination with CD206, CD209, and Dectin-1.

## 2. Materials and Methods

### 2.1. BD Patients

The patient population consisted of 13 patients with BD, who presented for the first time or were monitored at the Department of Rheumatology, Ajou University Hospital. Clinical characteristics and therapeutic histories of these patients are shown in Tables 1 and 2. According to the International Study Group for BD criteria, the presence of any two of the following symptoms, in addition to recurrent oral ulcerations, is considered to be sufficient for a BD diagnosis: recurrent genital ulceration, uveitis, large-vessel vasculitis, cutaneous erythema nodosum, arthritis, and/or a positive pathergy test. The disease severity score was followed by Behçet's disease current activity form 2006 (http://www.behcetdiseasesociety.org/behcetwsData/Uploads/files/BehcetsDiseaseActivityForm.pdf). The active BD patients with arthritis (*n* = 13, male 1, female 12, 46.3 ± 7.8 years) were enrolled and treated with adding or increasing corticosteroid or nonsteroidal anti-inflammatory drugs. Informed consent was obtained from patients prior to enrollment into the study. The healthy control (HC) group (*n* = 19, 37.7 ± 15.2 years) consisted of 6 male and 13 female participants. Blood sampling was done initially (active stage) and the follow-up after improving joint symptoms (inactive stage). Included disease control was patients with RA (*n* = 19, 30.4 ± 10.1 years). The medication for RA patients is shown in Supplementary Data (s-Table 1, in Supplementary Material available online at https://doi.org/10.1155/2017/7539529). This study was approved by the Institutional Review Board (IRB number BMR-SMP-13-398).

### 2.2. Flow Cytometric Analysis for Surface Markers

Collected peripheral blood mononuclear cells (PBMC) were treated with Ammonium-Chloride-Potassium solution for lysis of red blood cell and washed with buffered saline, after which, 1 × 10^6^ cells in each tube were incubated with allophycocyanin (APC) labeled anti-CD4 (Catalog number 17-0049-42, eBiosciences), fluorescein isothiocyanate (FITC) labeled anti-CD11b (Catalog number 11-0118-42, eBiosciences), PE-Cy7 labeled anti-CD11c (Catalog number 25-0116-41, eBiosciences), APC labeled anti-CD32 (Catalog number 17-0329-42, eBiosciences), PE labeled anti-CD209 (Catalog number 12-2099-42, eBiosciences), PerCP labeled anti-dectin-1 (Catalog number 1-46-9856-42, eBiosciences, San Diego, CA, USA), FITC labeled anti-CD8 (Catalog number 557085, BD Bioscience), and PE-Cy5 labeled anti-CD206 (Catalog number 551136, BD Pharmingen), for 30 minutes at 4°C. Same colors labeled antibodies were applied separately in different tubes. Isotype controls were applied also. The stained cells were then washed with buffered saline and analyzed by using a flow cytometer (FACSAria III; Becton Dickinson, San Jose, CA, USA) with ×10,000 cells. The fluorescence-activated cell sorting data was based on the gating in whole cells, granulocytes, monocytes, and lymphocytes and then applied to analyze for specific markers in gated populations. The data was repeatedly analyzed in triplicate.

### 2.3. Cytokine Assay

Peripheral blood was extracted in ethylenediaminetetraacetic acid containing test tubes and centrifuged at 3,500 rpm for 5 min at room temperature. The supernatant was stored at −80°C until required. Interleukin- (IL-) 18 (Catalog number BMS267/2CE, eBiosciences), IL-23 (Catalog number 88-7237-22, eBiosciences), and IL-17A (Catalog number BMS2017, eBiosciences) levels were measured in plasma using commercial enzyme-linked immunosorbent assay kits according to the manufacturer's instructions. For ELISA analysis, each sample was applied in duplicate wells and three times in different ELISA plates.

### 2.4. Statistical Analysis

All data shown represent the mean ± standard deviation. Statistical analysis of patients' data was performed using the Kruskal-Wallis Test and Bonferroni correction. Differences in frequencies of several cellular markers were determined using the Mann–Whitney *U* test. Correlations between levels and disease activity markers were evaluated with Spearman's correlation test. Statistical analyses were performed using SPSS for Windows software (ver. 23.0; IBM Corp., Armonk, NY, USA). A *p* value < 0.05 was considered to indicate statistical significance.

## 3. Results

### 3.1. Clinical Characteristics of the Patients

Clinical characteristics of the 13 patients with BD are summarized in Table 1. Blood sampling was performed twice at active stage of disease with arthritis (1st) and inactive stage without symptoms after medication (2nd). The disease severity scores and erythrocyte sedimentation rate (ESR) were significantly downregulated at 2nd sampling compared to the 1st sampling (*p* < 0.001, 0.038 resp.). The levels of C-reactive protein (CRP) were also downregulated, but not significant.  Table 2 shows the medication list at the time of blood sampling. Colchicine was used in 11 patients (84.6%), sulfasalazine in 6 patients (46.2%), and azathioprine in 3 patients (23.1%). And nonsteroidal anti-inflammatory drugs were used in 11 patients (84.6%).

### 3.2. The Frequencies of the PRRs on PBMC of BD Patients

**Figure 1:**
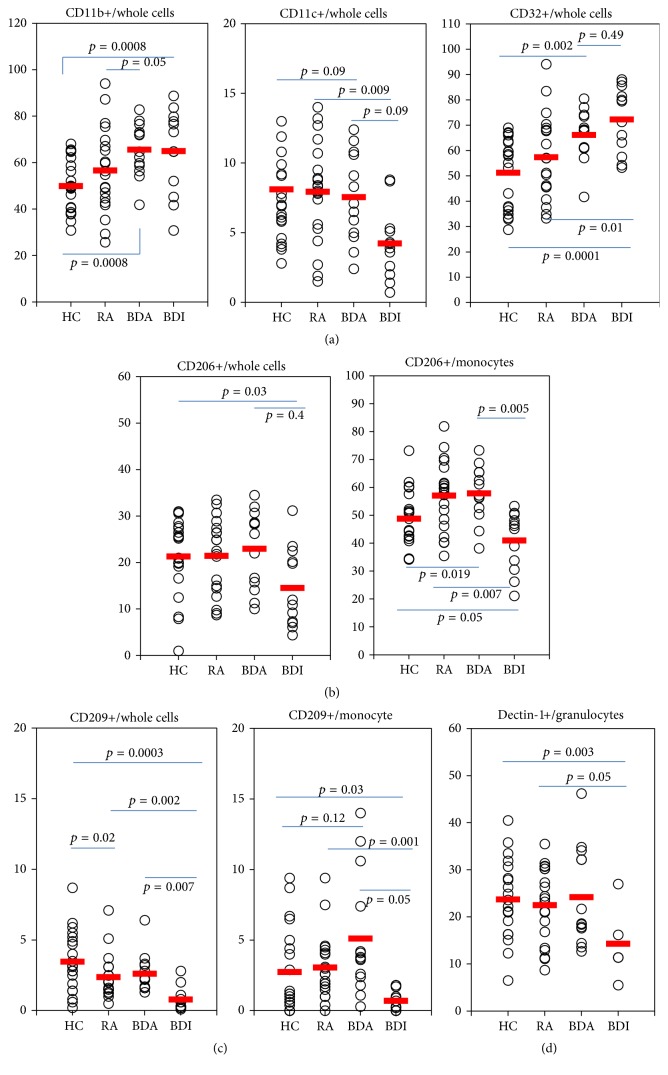
The frequencies of CD11b, CD11c, CD32, CD206, CD209, and dectin-1 positive cells in patients with active Behçet's disease (BDA), inactive BD (BDI), rheumatoid arthritis (RA), and healthy control (HC). The frequencies of CD11b, CD11c, and CD32 positive cells were analyzed in whole leukocytes. The frequencies of CD206, CD209, and dectin-1 positive cells were analyzed in whole leukocytes, granulocytes, and monocytes populations.

The frequencies of CD11b, CD11c, CD32, CD206, CD209, and Dectin-1 positive cells were analyzed on PBMC by flow cytometry after surface staining with antibodies. The frequencies of CD11b+ cells from whole leukocytes were significantly higher in patients with active BD (68.9 ± 11.2%) than in RA (55.6 ± 17.3%, *p* = 0.05) or HC (50.5 ± 12.0%, *p* = 0.0008). However, patients with inactive BD (66.4 ± 16.7%) showed similar frequencies to those with active BD (Figure 1(a)). The frequencies of CD11c+ cells in whole leukocytes were not significantly different among active BD, RA, and HC. After treatment, the frequencies of CD11c+ cells were decreased from 10.6 ± 3.0% in active BD to 7.1 ± 3.8% in inactive BD. CD32+ cells were significantly higher in active BD (66.1 ± 10.1%) than HC (51.3 ± 13.9%, *p* = 0.002) or RA (57.3 ± 16.9%). The differences between active BD and inactive BD for CD32+ cells were not significant (Figure 1(a)). The frequencies of CD206+ cells in whole leukocytes were downregulated to 14.5 ± 8.6% after treatment (inactive BD) compared to 23.0 ± 8.4% in active BD, even though not significant. In monocyte gating, the frequencies of CD206+ were significantly different between active BD (57.9 ± 9.4%) and inactive BD (41.0 ± 10.6%, *p* = 0.005). Also monocytes CD206+ in active BD was significantly high than HC (48.8 ± 10.5%, *p* = 0.019) (Figure 1(b)). The frequencies of CD209+ cells were significantly higher in active BD than inactive BD in whole leukocytes (2.4 ± 1.5% versus 0.8 ± 0.8%, *p* = 0.007). The frequencies of CD209+ cells were significantly decreased in inactive BD compared to active BD in monocytes (5.1 ± 4.1% versus 1.0 ± 0.7%, *p* = 0.05) (Figure 1(c)). The frequencies of Dectin-1+ cells in granulocytes were significantly lower in inactive BD (14.3 ± 8.0%) than HC (23.7 ± 8.4%, *p* = 0.003) (Figure 1(d)).

**Figure 2:**
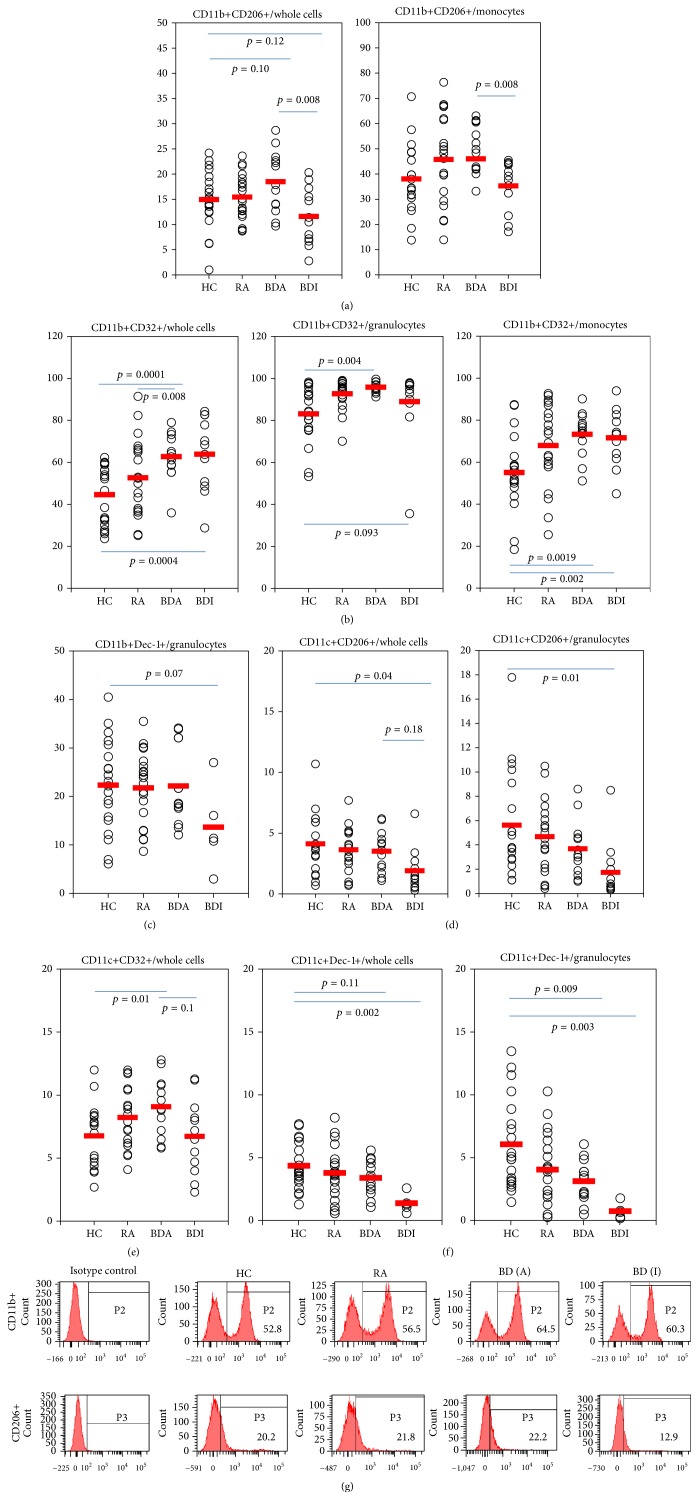
The frequencies of double positive cells in patients with active Behçet's disease (BDA), inactive BD (BDI), rheumatoid arthritis (RA), and healthy control (HC). CD11b+CD206+, CD11b+CD32+, CD11b+Dectin-1+, CD11c+CD206+, CD11c+CD32+, and CD11c+Dectin-1+ cells were analyzed in whole leukocytes, granulocytes, and monocyte (a–f). (g) Representative histograms of CD11b+ and CD206+ cells in HC, RA, BDA, and BDI.

Double positive cell frequencies were also analyzed (Figure 2). CD11b+CD206+ cells were significantly higher in active BD (18.5 ± 6.1%) than inactive BD (11.6 ± 5.7%, *p* = 0.008) in whole leukocytes and in monocytes (46.0 ± 9.7% versus 35.3 ± 10.1%, *p* = 0.008) (Figure 2(a)). CD11b+CD32+ cells were significantly higher in active BD (62.7 ± 11.0%) than HC (44.6 ± 14.0%, *p* = 0.0001) in whole leukocytes and in granulocytes (95.8 ± 2.4% versus 83.2 ± 13.2%, *p* = 0.004). However, these cells were not different between active BD (62.7 ± 11.0%) and inactive BD (63.9 ± 17.4%) in whole leukocytes. In monocytes, active BD and inactive BD were significantly higher than HC (*p* = 0.0019 and *p* = 0.002, resp.) (Figure 2(b)). CD11b+Dectin-1+ cells were lower in inactive BD (13.7 ± 8.8%) than HC (22.3 ± 9.4%) in granulocytes (Figure 2(c)). CD11c+CD206+ cells were significantly lower in inactive BD (1.9 ± 1.7%) than HC (4.1 ± 2.4%, *p* = 0.01) in whole cells and in granulocytes (1.7 ± 2.4% versus 5.6 ± 4.2%, *p* = 0.01) (Figure 2(d)). CD11c+CD32+ were higher in active BD (9.1 ± 2.3%) than HC (6.8 ± 2.4%, *p* = 0.01) in whole leukocytes. There was no difference in the frequencies of CD11c+CD32+ between active and inactive BD (Figure 2(e)). CD11c+Dectin-1+ cells were lower in inactive BD (1.4 ± 0.7%) than HC (4.4 ± 1.8%, *p* = 0.002) in whole leukocytes and in granulocytes (0.7 ± 0.6% versus 6.1 ± 3.6%, *p* = 0.003) (Figure 2(f)).  Figure 2(g) shows the representative histograms of fluorescence-activated cell sorting analysis.  Table 3 shows significantly different frequencies of PRR between active and inactive BD presented. Not significantly different frequencies of PRR between active and inactive BD showed in Supplementary Figure (s-Figure 1).

### 3.3. The Levels of IL-18, IL-23, and IL-17A in Plasma of BD Patients

**Figure 3:**
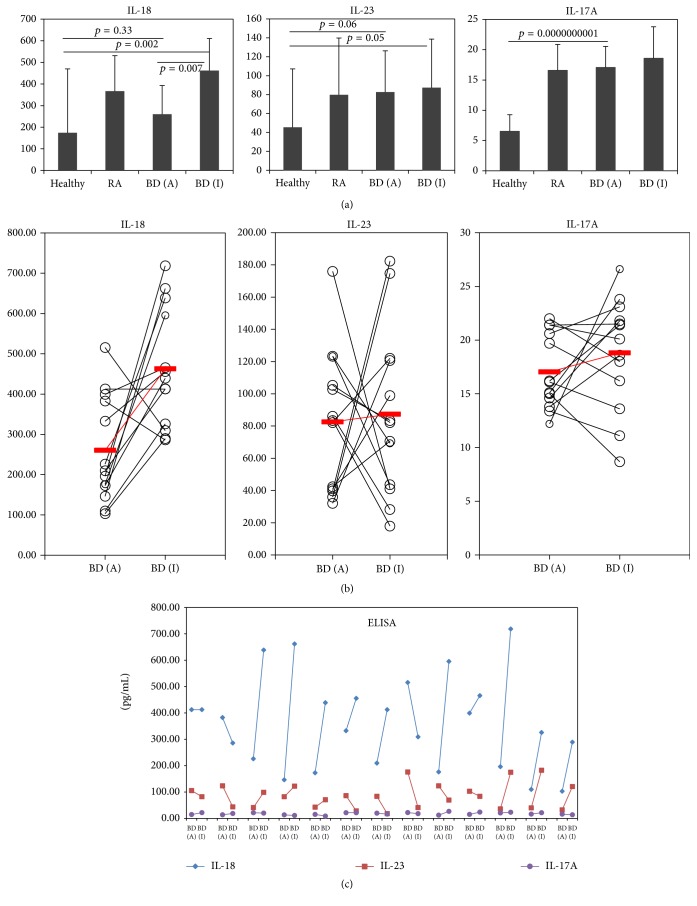
(a) Cytokine, IL-18, IL-23, and IL-17A expressions by enzyme-linked immunosorbent assay (ELISA) analysis in plasma of patients with active BD (BDA), inactive BD (BDI), rheumatoid arthritis (RA), and healthy control (HC). (b) The change of cytokine expression between with BDA and BDI in individual patients.

Figure 3 shows plasma levels of IL-18, IL-23, and IL-17A in active and inactive BD patients, RA patients, and HC. After treatment, the expression levels of IL-18 increased to 462.3 ± 148.0 pg/mL in inactive BD from 260.1 ± 132.5 pg/mL in active BD (*p* = 0.007). IL-23 levels were not significantly different between active BD (82.6 ± 43.7 pg/mL) and inactive BD (87.4 ± 51.7 pg/mL). IL-17A levels were also similar between active BD (17.0 ± 3.5 pg/mL) and inactive BD (18.8 ± 5.2 pg/mL) (Figure 3(a)). Even though the average levels of IL-23 and IL-17A from all enrolled patients did not change much after treatment, the levels of each individual patient were increased or decreased as shown in Figure 3(b).  Figure 3(c) shows three cytokine levels of each individual patient analyzed before and after treatment. The levels of cytokines were not changed in the same direction after improvement of patients.

### 3.4. The Correlations between Disease Activity Markers and Frequencies of PRR Expressing Cells in BD Patients

The correlation between disease severity score and PRR, such as CD11b in granulocytes (*r* = 0.504, *p* = 0.009); CD11c in whole leukocytes (*r* = 0.485, *p* = 0.012), granulocytes (*r* = 0.427, *p* = 0.03), and lymphocytes (*r* = 0.649, *p* = 0.001); CD206 in whole leukocytes (*r* = 0.412, *p* = 0.036), granulocytes (*r* = 0.438, *p* = 0.025), monocytes (*r* = 0.509, *p* = 0.008), and lymphocytes (*r* = 0.518, *p* = 0.007); CD209 in whole leukocytes (*r* = 0.642, *p* = 0.001), granulocytes (*r* = 0.48, *p* = 0.013), monocytes (*r* = 0.615, *p* = 0.001), and lymphocytes (*r* = 0.584, *p* = 0.002); and Dectin-1 in granulocytes (*r* = 0.46, *p* = 0.047) and lymphocytes (*r* = 0.668, *p* = 0.002) showed significance. The plasma levels of IL-17A also showed significant correlations to CD206 in lymphocytes (*r* = −0.409, *p* = 0.038); CD209 in whole leukocytes (*r* = −0.5, *p* = 0.009), granulocytes (*r* = −0.512, *p* = 0.008), monocytes (*r* = −0.52, *p* = 0.006), and lymphocytes (*r* = −0.477, *p* = 0.014) (Table 4). Other disease activity markers, such as leukocyte, ESR, CRP, cytokine IL-23, and IL-18 did not show a significant correlation.

### 3.5. Correlation between the Frequencies of Each PRR Marker in BD Patients

The correlation analysis was also applied to each surface marker of PRR in whole leukocytes (Table 5). The frequencies of CD11b were correlated to the frequencies of CD32 (*r* = 0.896, *p* < 0.001). The frequencies of CD11c were correlated to the frequencies of CD206 (*r* = 0.83, *p* < 0.001) and CD209 (*r* = 0.677, *p* < 0.001). The frequencies of CD32 were negatively correlated with the frequencies of CD206 (*r* = −0.439, *p* = 0.025). The frequencies of CD206 were correlated positively with the frequencies of CD209 (*r* = 0.679, *p* < 0.001) and Dectin-1 (*r* = 0.801, *p* = 0.001).

## 4. Discussion

This study has presented the significantly different expression levels of the frequencies for PRR between BD patients and HC. The frequencies of CD11b, CD11c, CD32, CD206, CD209, and Dectin-1 positive cells were differently expressed on whole leukocytes, granulocytes, monocytes, or lymphocytes among active BD, inactive BD, and HC. The frequencies of CD206 and CD209 showed significant correlation with plasma levels of IL-17A. Specifically, the frequencies of CD11b, CD11c, CD32, CD206, CD209, and Dectin-1 positive cells were correlated significantly to the disease severity score of BD patients.

It is well known PRR plays critical roles in pathogen recognition and linking to the generation of adaptive immunity [10]. Toll-like receptor, a family of PRR, has been revealed in BD [11–14]. However, C-type lectin receptors, another family of PRR, CD206, CD209, and Dectin-1, can be found in a few cases in BD patients until now. Downregulation/deficiency of C-type lectin domain family 12, member A (CLEC12A), a C-type lectin-like pattern recognition receptor, is associated with hyperinflammatory responses. Patients with severe forms of Behçet's disease underexpress CLEC12A with respect to patients with mild forms of the disease [15]. Mannose-binding lectin gene-2 polymorphisms and serum mannose-binding lectin levels are associated with the production of high levels of MBL in Behçet's disease. Dectin-1 is upregulated on immune cells such as macrophages and neutrophils in inflammatory bowel disease [16]. Dectin-1 plays a critical role in experimental autoimmune uveitis development by Freund's Complete Adjuvant/interphotoreceptor retinoid-binding protein mixture at the induction phase. Uveitis is one of the major symptoms of BD in patients. Anti-dectin-1 antibody treatment can prevent the development of experimental autoimmune uveitis [17]. In BD patients, the correlation with Dectin-1 was not reported. CD209 promote CD3-activated T cells to produce IL-2 and strengthen the interaction of T cell receptor-antigen presenting cell and enhance immune response [18]. CD209 was reported in secondary progressive multiple sclerosis [19] and Crohn's disease [20]; however, the correlation was not studied in BD. Our study showed the correlation of several PRR (CD11b, CD11c, CD32, CD206, CD209, and dectin-1) in the pathogenesis of BD.

The mean fluorescence intensity of CD11b on granulocytes was higher in BD patients with heart diastolic dysfunction than those of controls [21]. The mean fluorescence intensity of CD11b in CD8 bright cells was also higher in BD patients with uveitis than controls or BD without uveitis [22]. Similar to previous reports, our study showed that BD patients with arthritis had higher frequencies of CD11b+ cells than disease control RA patients and the HC. After improvement with treatment, the frequencies of CD11b+ cells of inactive BD were not changed.

Single nucleotide polymorphisms of CD11c have been suggested as susceptibility loci in Korean BD patients with cutaneous symptoms [23] and in Chinese Han population BD patients with eye symptoms [24]. The frequencies and protein expression levels of CD11c+ cells were significantly increased in the CD4+ and CD8+ cell populations of active BD patients with cutaneous symptoms compared to those in the HC. In BD patients with arthritis, from our study, CD11c+ cells showed similar frequencies to HC. Inactive BD patients showed downregulation of CD11c+ cells compared to active BD patients though the difference was not significant. This difference can be from the different immune responses between BD cutaneous symptoms and BD arthritic symptoms, even though 20% of enrolled patients have both cutaneous and arthritic symptoms in each report.

Until now, there were no papers regarding CD32 in BD patients. This is the first data describing CD32 in BD patients. In RA patients, CD32 on natural killer (NK) cells were significantly lower than HCs [25]. However, in our results the frequencies of CD32+ cells on whole leukocytes were higher in active BD patients with arthritis than HCs. In RA patients as a disease control, CD32 expressions were not downregulated as compared to HCs. Inactive BD patients were not different as compared to active BD patients with arthritis. It was reported that CD32 participated in the stimulation of plasmacytoid DC by immune complex combined with anti-DNA antibody in systemic lupus erythematosus [26]. CD32 seems to be involved in the induction of inflammatory responses, but still not enough for defining of the role in inflammation of BD.

The correlation of CD206 and BD has also been rarely reported. There is only one paper for CD206 in BD patients with cutaneous symptoms [27]. CD206 was highly expressed with significance in active BD patients with cutaneous symptoms (49.7 ± 35.2%) compared to inactive BD patients (4.7 ± 3.1%) (*p* = 0.007) and HCs (7.4 ± 0.8%) (*p* = 0.02). It is known that CD206 facilitates viral infections such as Dengue virus [28], hepatitis B virus [29], lymphadenopathy associated virus [30], and human immunodeficiency virus [31]. In collagen induced arthritis, CD206 was seen in the inflamed joints by micro-computed tomography imaging [32]. CD206^+^ macrophages were significantly induced in patients with mucosal healing after treatment with infliximab [33]. In the mixed lymphoid reaction, the antibodies against tumor necrosis factor induced CD206+ macrophages. CD206+ macrophages had an immunosuppressive phenotype showing inhibited proliferation of activated T cells [34]. In our results, CD206+ PBMC were downregulated after treatment compared to before treatment in BD patients.

The correlation of CD209 and BD has not been reported at all. Our present study has shown the frequencies of CD209+ cells were significantly decreased in whole leukocytes and monocytes populations after improvement compared to an active symptomatic state. In juvenile idiopathic arthritis, CD209+ cells were increased in peripheral blood and accumulated in synovial fluid [35]. CD209+ cells were also higher in RA synovium than osteoarthritis synovium [36]. CD209^+^CD14^+^ cells were significantly increased in the lesion of lamina propria of Crohn's disease [37]. This subset might be involved in the pathogenesis of Crohn's disease [38].

Dectin-1 has been known to play a role in antifungal immunity [39] for the protection from* Candida albicans* infection [40] and is involved in the production of arachidonic acid metabolites [41] and DC maturation [42]. Dectin-1 recognizes *β*-glucans and carbohydrates found in some bacteria and may also recognize other molecules such as endogenous ligand on T cells [43]. Dectin-1 expression is found on myeloid DC, monocytes, macrophages, and B cells [44]. In a genetically arthritis susceptible mouse model, Dectin-1 plays a role for the induction of arthritis [45]. In our experiment, the frequencies of Dectin-1+ cells in granulocytes populations were downregulated after treatment compared to active BD arthritis. Dectin-1 signaling increases the phagocytic activity of neutrophils through upregulation of CD11b expression [46] and increased the production of IL-17A. Dectin-1-dependent IL-17A production is mediated by CD11b+ neutrophils in an IL-23 dependent manner [47]. Our data has shown CD11b+Dectin-1+ granulocytes were downregulated after improvement of BD arthritis though the significance was not different. This is the first data for the Dectin-1 expression in BD.

CD11b+CD206+ macrophages play critical roles in the progression of hepatitis C virus induced inflammatory liver diseases [48]. In our results, the frequencies of CD11b+CD206+ in whole leukocytes and monocytes were downregulated with improvement of BD arthritis. Also, CD11b+CD32+ cell populations in whole leukocytes and monocytes were higher in BD patients compared to HC irrespective of arthritis activity.

With flow cytometric data, we can conclude the frequencies of CD11b+ and CD32+ cells show similar levels in active and inactive BD. CD11c+, CD206+, CD209+, and Dectin-1 were downregulated after improvement. This means higher levels of CD11b+ and CD32+ cells are correlated to the development of BD arthritis. CD11c+, CD206+, CD209+, and Dectin-1 are related to the regulation of BD symptom. For the therapeutic target, CD11c+, CD206+, CD209+, and Dectin-1 can be more reliable candidates for BD arthritis. This can be a direction for future experimental plans.

Several papers have shown higher levels of IL-18 in BD patients than HC [49, 50]. IL-18 with IL-12 or IL-15 enhances the Th1 response, while IL-18 without IL-12 can stimulate Th2 responses [51]. IL-18 with IL-2 or IL-4 enhances the production of IL-4 and IL-13 [52]. In our data, inactive BD patients showed significantly higher IL-18 levels than active BD patients. In BD patients with arthritis, increased IL-18 in improved stage may have a role for Th2 shift function with IL-4 or IL-2, even though we did not show the levels of IL-4 or IL-2.

The serum levels of IL-23 are elevated more in BD patients with active uveitis than HC. BD patients with active uveitis significantly upregulated IL-23 as compared with BD patients without active uveitis [53]. In our data, the levels of IL-23 were higher in BD patients with arthritis than HC, though the level of IL-23 was not different between active and inactive BD patients. IL-17 was also significantly higher in active BD patients than HC. However, active and inactive BD patients with arthritis showed similar levels of IL-17. Several papers already reported BD patients with uveitis showed similar levels of IL-17A between active and inactive stage [54, 55]. IL-18 gene polymorphism was reported by 5 papers in Turkish, Egyptian, and Korean. These 5 papers did not include the protein levels of IL-18. Oztas et al. reported the increased serum IL-18 protein expression compared to healthy control, but they did not show the comparison between active BD and inactive BD [56]. Musabak et al. reported the serum levels of IL-18 were increased in patients with inactive disease than active disease even though the difference was not significant [57]. They mentioned Th1 activation and subclinical inflammation persists during the inactive period of the disease. In our result, similarly to the previous reports, plasma IL-18 levels were higher in active BD than HC. Also, IL-18 in inactive BD was higher than active BD. According to Habibagahi et al., serum levels of IL-23 were higher in BD with uveitis [58]. IL-23 and IL-17 were higher in BD with active uveitis than BD with inactive uveitis [53]. Among this patients population, 33.3% of patients had arthritis. According to Emiroglu et al., serum IL-17A levels were not different between active BD and inactive BD [55]. This study included 57.9% articular involvement in cutaneous BD symptomatic patients. They enrolled different populations with and without symptoms instead of chasing the same patients. The expression was similar to our data. In our data, the average levels of IL-23 and IL-17 were similar between pretreatment and posttreatment; however, the changes of expression levels are diverse in each patient.

In conclusion, the CD11b+ and CD32+ circulating cells were significantly higher in active BD patients with arthritis than HC. CD11c+ and CD209+ cells were significantly decreased after improvement of arthritis in BD patients. Disease severity scores were significantly correlated to the frequencies of CD11b+, CD11c+, CD206+, CD209+, and Dectin-1+ cells. Plasma IL-17A expression levels were also correlated with the frequencies of CD206+ and CD209+ cells. CD206 in monocytes, CD209 in whole cells and monocytes, and CD11b+CD206+ in whole cells and monocytes can be disease activity markers for the improvement of BD arthritis.

## Supplementary Material

s-Table 1. Treated medications when the blood sampling in Rheumatoid arthritis patients.
s-Figure 1: The frequencies of CD206, CD209 and dectin-1 positive cells in patients with active Behçet's disease (BDA), inactive BD (BDI), rheumatoid arthritis (RA), and healthy control (HC). The frequencies of CD206 and CD209 positive cells were analyzed in granulocytes populations. The frequencies of dectin-1 positive cells were analyzed in whole leukocytes and monocytes populations. The frequencies of double positive cells in patients with active Behçet's disease (BDA), inactive BD (BDI), rheumatoid arthritis (RA), and healthy control (HC). CD11b+CD206+, CD11b+Dec-1+, CD11c+CD206+, CD11c+CD32+ and CD11c+Dec-1+ cells were analyzed in whole leukocytes, granulocytes and monocytes.

## Figures and Tables

**Table 1:** Clinical characteristics of the 13 patients with Behçet's disease.

Patients	Sex	Age	OU	GU	Arthritis	EN	Severity score		Leukocyte		ESR		CRP	
							1st	2nd	1st	2nd	1st	2nd	1st	2nd
1	F	55	+	−	+	+	4	0	7300	9500	95	15	4.66	0.19
2	F	36	−	−	+	+	3	0	3900	4300	57	40	0.4	0.17
3	F	59	−	−	+	−	2	0	5900	7200	21	14	0.03	0.03
4	M	41	−	−	+	−	2	2	13900	10800	43	19	7.92	0.73
5	F	50	−	−	+	−	2	2	5900	7700	6	10	0.03	0.22
6	F	49	+	−	+	+	4	2	6100	6500	4	2	0.2	0.04
7	F	45	+	+	+	+	5	2	10800	5500	5	7	0.03	0.02
8	F	41	+	−	+	−	4	2	5600	5400	21	14	0.12	0.02
9	F	47	−	−	+	−	2	0	8500	6700	13	35	0.27	1.11
10	F	42	+	−	+	−	3	2	7600	6600	18	6	0.07	0.02
11	F	32	−	−	+	+	3	2	6400	5000	70	39	0.14	0.04
12	F	51	+	+	+	−	4	2	5900	5600	71	43	2.93	0.31
13	F	54	−	−	+	−	2	0	5400	6700	43	19	0.59	0.4
Average		46.3					3.07	1.23	7169.2	6730.8	35.9	20.4	1.34	0.25
SD		7.8					1.03	1.01	2634.8	1801.0	29.7	14.1	2.42	0.33
*p* value							0.00008		0.46		0.038		0.11	

OU: oral ulcer, GU: genital ulcer, EN: erythema nodosum, ESR: erythrocyte sedimentation rate, CRP: C-reactiveprotein, 1st: first blood sampling, 2nd: second blood sampling, SD: standard deviation.

**Table 2:** Medications during the blood sampling in Behçet's disease patients with arthritis.

Patients	Colchicine		Steroid		AZP		Bucillamine		HCQ		SZP		NSAIDs	
	1st	2nd	1st	2nd	1st	2nd	1st	2nd	1st	2nd	1st	2nd	1st	2nd
1	+	+	+	+	+	−	−	−	−	−	−	+	+	+
2	−	−	−	−	+	+	−	−	−	−	+	+	−	−
3	+	+	+	−	−	−	−	−	−	−	+	+	+	+
4	+	+	+	+	−	+	+	+	−	−	−	−	+	+
5	+	+	−	+	−	−	−	−	−	−	+	+	−	−
6	+	+	+	−	−	−	−	−	+	+	−	−	+	+
7	+	+	+	+	−	−	−	−	+	+	+	+	+	+
8	+	+	+	−	−	−	−	+	−	−	+	−	+	−
9	+	+	+	+	−	−	−	−	+	+	−	−	+	+
10	+	+	+	+	−	−	−	−	+	+	−	−	+	−
11	−	−	+	+	+	+	−	−	−	−	−	−	+	+
12	+	+	+	+	−	−	−	−	−	−	+	+	+	+
13	+	+	+	+	−	−	−	−	+	+	−	−	+	−

AZP: azathioprine, HCQ: hydroxychloroquine, SZP: sulphasalazine, NSAIDs: nonsteroidal anti-inflammatory drugs.

**Table 3:** The significant changes of PRR between active and inactive Behçet's disease patients.

Markers	Cell population	Active BD	Inactive BD	*p* value
CD206+	Monocyte	57.9 ± 9.4	41.0 ± 10.6	0.005
CD209+	Whole leukocyte	2.4 ± 1.5	0.8 ± 0.8	0.007
CD209+	Monocyte	5.1 ± 4.1	1.0 ± 0.7	0.05
CD11b+CD206+	Whole leukocyte	18.5 ± 6.1	11.6 ± 5.7	0.008
CD11b+CD206+	Monocyte	46.0 ± 9.7	35.3 ± 10.1	0.008

**Table 4:** Correlations between the frequencies of pattern recognition receptors (PRR) expressing cells and disease activity markers in patients with Behçet's disease.

Disease activity markers	Correlation coefficient, *r* (*p* value)													
	CD11b granulocyte	CD11c whole	CD11c granulocyte	CD11c lymphocyte	CD206 whole	CD206 granulocyte	CD206 monocyte	CD206 lymphocyte	CD209 whole	CD209 granulocyte	CD209 monocyte	CD209 lymphocyte	Dectin granulocyte	Dectin lymphocyte
Disease severity score	**0.504 (0.009)**	**0.485 (0.012)**	**0.427 (0.03)**	**0.649 (<0.001)**	**0.412 (0.036)**	**0.438 (0.025)**	**0.509 (0.008)**	**0.518 (0.007)**	**0.642 (<0.001)**	**0.48 (0.013)**	**0.615 (0.001)**	**0.584 (0.002)**	**0.46 (0.047)**	**0.668 (0.002)**

Leukocyte	0.055 (0.79)	−0.158 (0.441)	−0.162 (0.43)	−0.078 (0.704)	−0.137 (0.503)	−0.152 (0.457)	−0.118 (0.566)	−0.193 (0.346)	−0.167 (0.414)	−0.201 (0.325)	−0.047 (0.82)	0.063 (0.761)	−0.318 (0.185)	−0.139 (0.57)

ESR	0.094 (0.649)	0.008 (0.971)	−0.017 (0.935)	0.105 (0.609)	0.149 (0.467)	0.019 (0.927)	0.233 (0.252)	0.161 (0.433)	0.241 (0.235)	0.155 (0.449)	0.061 (0.769)	0.323 (0.108)	0.185 (0.449)	0.207 (0.396)

CRP	0.248 (0.223)	−0.159 (0.438)	−0.212 (0.298)	0.016 (0.939)	−0.071 (0.73)	−0.203 (0.32)	0.131 (0.525)	−0.001 (0.996)	0.077 (0.707)	0.025 (0.902)	0.04 (0.845)	0.25 (0.218)	−0.068 (0.781)	0.272 (0.26)

IL-17A	−0.287 (0.155)	−0.267 (0.188)	−0.216 (0.29)	−0.321 (0.109)	−0.337 (0.092)	−0.304 (0.131)	−0.244 (0.23)	**−0.409 (0.038)**	**−0.5 (0.009)**	**−0.512 (0.008)**	**−0.52 (0.006)**	**−0.477 (0.014)**	−0.322 (0.178)	−0.16 (0.513)

IL-23	0.156 (0.446)	−0.187 (0.36)	−0.169 (0.408)	−0.036 (0.863)	−0.11 (0.591)	−0.045 (0.828)	−0.004 (0.984)	0.057 (0.783)	0.017 (0.933)	0.041 (0.843)	−0.08 (0.698)	−0.019 (0.926)	0.113 (0.646)	0.049 (0.841)

IL-18	−0.045 (0.829)	−0.047 (0.818)	−0.036 (0.863)	−0.1 (0.628)	−0.236 (0.246)	−0.195 (0.339)	−0.207 (0.311)	−0.248 (0.222)	−0.211 (0.301)	−0.218 (0.285)	−0.117 (0.569)	−0.184 (0.367)	−0.192 (0.431)	−0.164 (0.501)

**Table 5:** Correlations between each pattern recognition marker in whole leukocytes of patients with Behçet's disease.

	Correlation coefficient, *r* (*p* value)				
	CD11c	CD32	CD206	CD209	Dectin
CD11b	−0.159 (0.438)	**0.896 (<0.001)**	−0.225 (0.268)	−0.128 (0.534)	0.05 (0.839)
CD11c		−0.357 (0.074)	**0.83 (<0.001)**	**0.677 (<0.001)**	**0.724 (<0.001)**
CD32			−**0.439 (0.025)**	−0.252 (0.214)	−0.157 (0.521)
CD206				**0.679 (<0.001)**	**0.801 (<0.001)**
CD209					0.334 (0.163)
