# Supplementary material for: The Correlation of CD206, CD209, and Disease Severity in Behçet's Disease with Arthritis
Source: Mediators Inflamm. 2017 Mar 9;2017:7539529. doi: 10.1155/2017/7539529 (PMC5362722; doi:10.1155/2017/7539529)
Supplement: Supplementary file 1 — s-Table 1. Treated medications when the blood sampling in Rheumatoid arthritis patients. s-Figure 1: The frequencies of CD206, CD209 and dectin-1 positive cells in patients with active Behçet's disease (BDA), inactive BD (BDI), rheumatoid arthritis (RA), and healthy control (HC). The frequencies of CD206 and CD209 positive cells were analyzed in granulocytes populations. The frequencies of dectin-1 positive cells were analyzed in whole leukocytes and monocytes populations. The frequencies of double positive cells in patients with active Behçet's disease (BDA), inactive BD (BDI), rheumatoid arthritis (RA), and healthy control (HC). CD11b+CD206+, CD11b+Dec-1+, CD11c+CD206+, CD11c+CD32+ and CD11c+Dec-1+ cells were analyzed in whole leukocytes, granulocytes and monocytes. [file 7539529.f1.docx]

s-Table 1. Treated medications when the blood sampling in Rheumatoid arthritis patients

| RA patients | Medication |
| --- | --- |
| 1 | Methotrexate 10mg weekly, hydroxychloroquine 300mg |
| 2 | Methotrexate 10mg weekly, hydroxychloroquine 300mg, prednisolone 2.5mg, meloxicam 7.5mg |
| 3 | Sulfasalazine 1000mg, hydroxychloroquine 300mg, prednisolone 5mg, meloxicam 15mg |
| 4 | Methotrexate 7.5mg weekly, hydroxychloroquine 200mg, leflunomide 20mg, methylprednisolone 1mg, meloxicam 7.5mg |
| 5 | Methotrexate 15mg weekly, hydroxychloroquine 300mg, deflazacort 6mg, aceclofenac 100mg |
| 6 | Methotrexate 12.5mg weekly, bucilamine 200mg, methylprednisolone 1mg |
| 7 | Methotrexate 12.5mg weekly, hydroxychloroquine 200mg, mizoribine 50mg, methylprednisolone 2mg, meloxicam 7.5mg |
| 8 | Sulfasalazine 1000mg, hydroxychloroquine 300mg, aceclofenac 200mg |
| 9 | Hydroxychloroquine 200mg, aceclofenac 200mg |
| 10 | Hydroxychloroquine 200mg, zaltoprofen 80mg |
| 11 | Sulfasalazine 1000mg |
| 12 | Hydroxychloroquine 300mg, methylprednisolone 4mg, aceclofenac 200mg |
| 13 | Hydroxychloroquine 300mg, meloxicam 7.5mg |
| 14 | Methotrexate 7.5mg weekly, hydroxychloroquine 200mg, methylprednisolone 1mg, pelubiprofen 30mg |
| 15 | Methotrexate 7.5mg weekly, hydroxychloroquine 200mg, methylprednisolone 2mg, aceclofenac 100mg |
| 16 | Aceclofenac 100mg |
| 17 | Methotrexate 7.5mg weekly, sulfasalazine 1000mg, methylprednisolone 2mg, pelubiprofen 60mg |
| 18 | Hydroxychloroquine 200mg, bucillamine 200mg, meloxicam 15mg |
| 19 | Methotrexate 15mg weekly, methylprednisolone 2mg |
