# Supplementary material for: The Correlation of CD206, CD209, and Disease Severity in Behçet's Disease with Arthritis
Source: Mediators Inflamm. 2017 Mar 9;2017:7539529. doi: 10.1155/2017/7539529 (PMC5362722; doi:10.1155/2017/7539529)
Supplement: Supplementary file 2 [file 7539529.f2.docx]

HC RA BDA BDI

P=0.134

HC RA BDA BDI

P=0.1

P=0.07

P=0.24

HC RA BDA BDI

P=0.002

P=0.08

P=0.007

HC RA BDA BDI

s-Figure 1.

HC RA BDA BDI

P=0.09

P=0.007

HC RA BDA BDI

HC RA BDA BDI

P=0.13

HC RA BDA BDI

HC RA BDA BDI

HC RA BDA BDI

HC RA BDA BDI

P=0.001

P=0.04

P=0.04

s-Figure 1: The frequencies of CD206, CD209 and dectin-1 positive cells in patients with active Behçet’s disease (BDA), inactive BD (BDI), rheumatoid arthritis (RA), and healthy control (HC) by flow cytometry. CD11b+CD206+, CD11b+Dec-1+, CD11c+CD206+, CD11c+CD32+ and CD11c+Dec-1+ double positive cells were analyzed in whole leukocytes, granulocytes and monocytes populations.
